# Delayed rupture of traumatic anterior cerebral artery A4 segment aneurysm: A case report

**DOI:** 10.1097/MD.0000000000033974

**Published:** 2023-06-09

**Authors:** Yu Shi, Yihang Sui, Kai Chen, Wenzhang Luo, Tianyu Zhang, Changren Huang, Kunyang Bao

**Affiliations:** a Department of Neurosurgery, The Affiliated Hospital of Southwest Medical University, Luzhou, Sichuan, China; b Department of Neurology, The Affiliated Hospital of Southwest Medical University, Luzhou, Sichuan, China; c Neurosurgical Clinical Research Center of Sichuan Province, Luzhou, China; d Laboratory of Neurological Diseases and Brain Functions, The Affiliated Hospital of Southwest Medical University, Luzhou, China.

**Keywords:** arteriae cerebri anterior, case report, cerebral angiography, computed tomographic angiography, traumatic intracranial aneurysm

## Abstract

Delayed rupture of traumatic intracranial aneurysms is relatively rare, and traumatic anterior A4 segment aneurysms may be associated with trauma to the cerebral falx. The mortality rate in patients with delayed traumatic rupture of intracranial aneurysms is>50%. Therefore, early diagnosis and treatment are critical. Herein, we present a case of a patient who did not have an intracranial aneurysm on computed tomographic angiography (CTA) after admission. Subsequently, the patient consciousness deteriorated, and CTA revealed aneurysm and bleeding.

**Patient concerns:**

A 55-year-old man fell from a 3-meter-high truck and was unconscious. During the following few hours, the gradually regained consciousness. No intracranial aneurysms were found on CTA of the patient head immediately after admission.

**Diagnoses:**

The final diagnosis was delayed rupture of traumatic intracranial aneurysms.

**Interventions:**

The patient underwent endovascular and symptomatic treatments.

**Outcomes:**

The patient gradually recovered and was referred to the rehabilitation department for further treatment.

**Lessons:**

Considering the catastrophic consequences of the disease, we should review CTA or digital subtraction angiography many times after admission, and take appropriate surgical procedures in time.

## 1. Introduction

Traumatic intracranial aneurysms are uncommon, accounting for <1% of all cerebral aneurysms. They may occur after blunt or penetrating head injury.^[1]^ The mortality rate of traumatic intracranial aneurysms is high.^[2]^ Traumatic intracranial aneurysms with delayed rupture are rarer. We report the case of a patient with no intracranial aneurysm found on computed tomographic angiography (CTA) after trauma. A few days later, the intracranial aneurysm ruptured.

## 2. Case report

**Figure 1.**
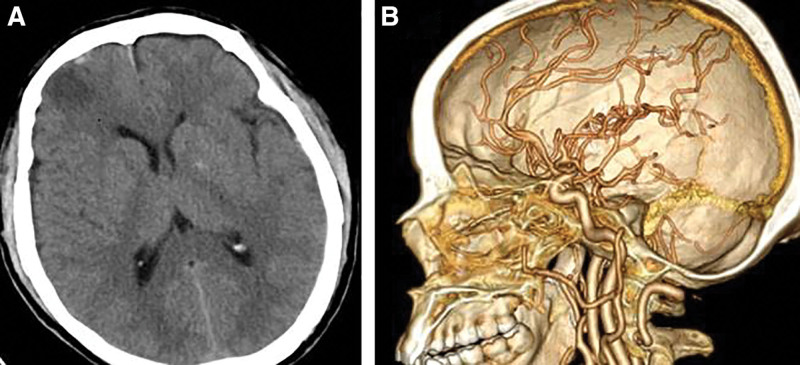
(A) Computed tomography scan of the bilateral frontal and temporal lobe brain contusion with a small hematoma, left temporal subdural hematoma, and left temporal parietal bone linear fracture. (B) Computed tomographic angiography showing no right anterior cerebral artery aneurysms.

**Figure 2.**
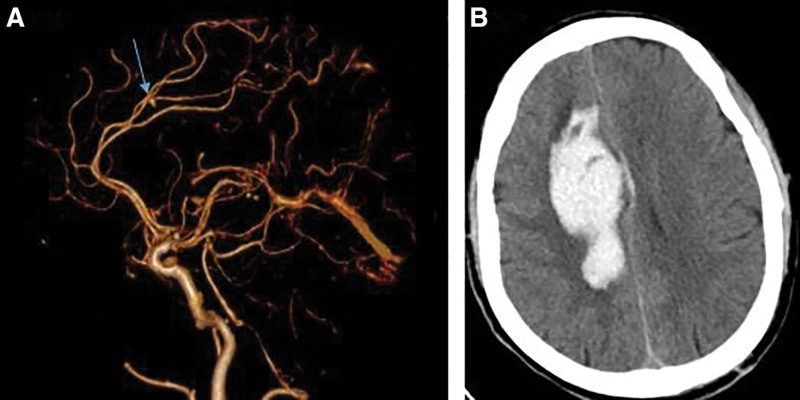
(A) CTA showed right anterior cerebral artery A4 segment aneurysm, about 0.3 cm × 0.3 cm × 0.4 cm. (B) Bilateral frontal and temporal lobe brain contusion; cerebral hemorrhage in the right corpus callosum and cingulate gyrus, left temporal parietal bone linear fracture with left temporal subdural hematoma; subarachnoid hemorrhage. CTA = computed tomographic angiography.

**Figure 3.**
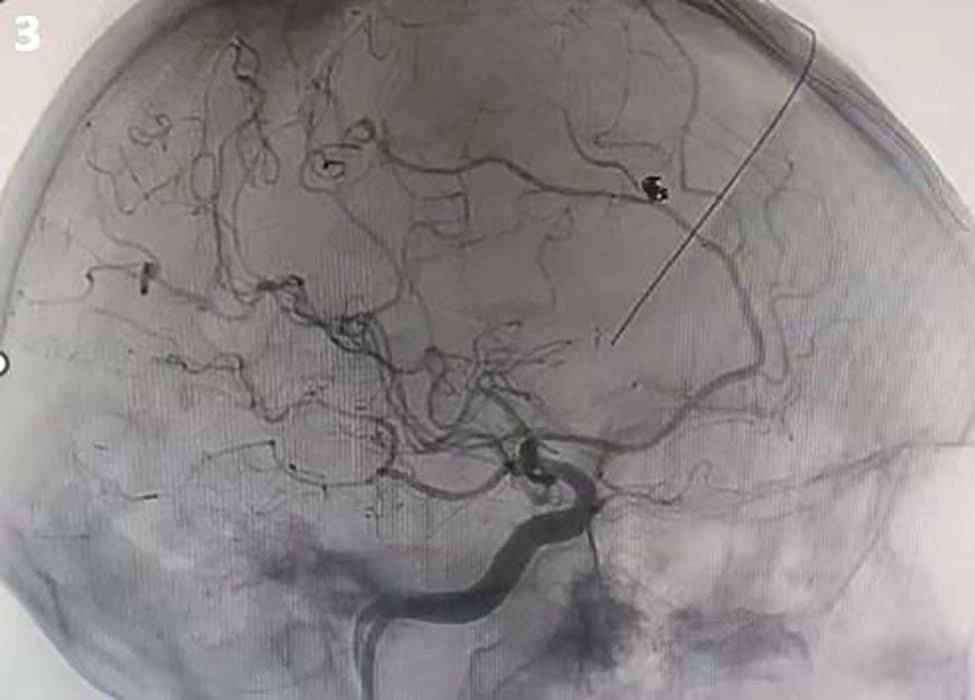
Cerebral angiography after stent-assisted embolization showed good embolization of intracranial aneurysms.

**Figure 4.**
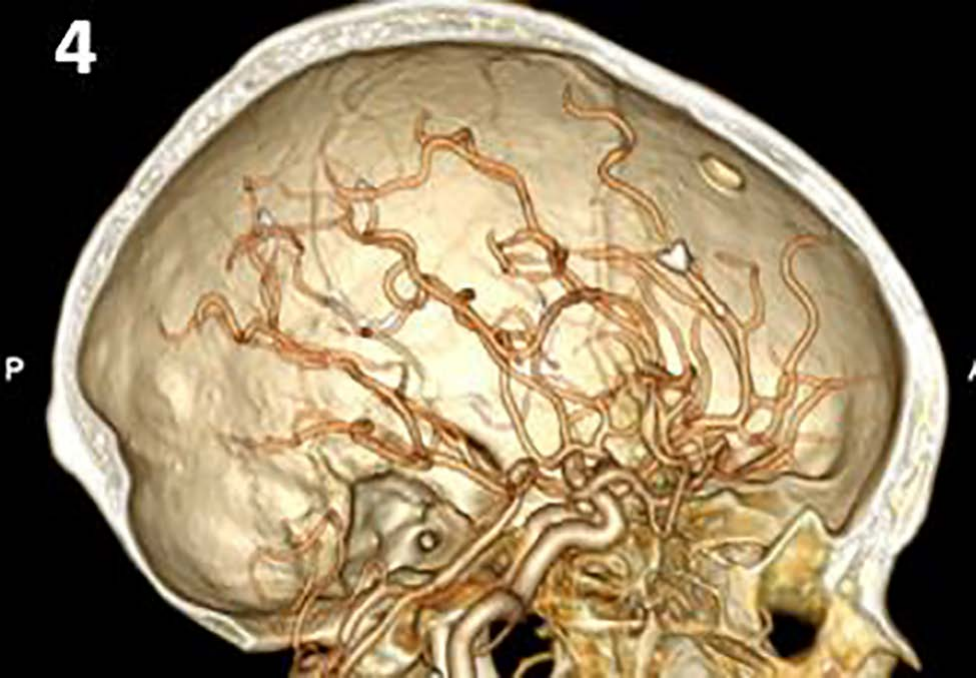
One mo later, the patient ‘s head CTA. CTA = computed tomographic angiography.

A 55-year-old man fell from a 3-meter-high truck and was unconscious. The patient had no history of disease. During the following few hours, the gradually regained consciousness. when admitted to the head of the hospital showed frontal lobe and temporal lobe brain contusion with small hematoma formation, left temporal subdural hematoma, and left temporal parietal bone fracture (Fig. 1A). No obvious right anterior cerebral artery abnormalities (Fig. 1B). After 7 days, the patient sudden consciousness deteriorated and he could not respond. CTA scan showed 0.3 × 0.3 × 0.4 aneurysms in the A4 segment of the right anterior cerebral artery (Fig. 2A), and cerebral hemorrhage in the right corpus callosum and cingulate gyrus (Fig. 2B). Immediate craniotomy for hematoma removal and decompressive craniectomy were performed. During the surgery, the vascular wall of the A4 segment of the anterior cerebral artery was damaged, forming a dissecting aneurysm. Plastic clipping was difficult; the aneurysm was wrapped, and the external ventricular drainage system was retained. The patient was gradually awakened. After 13 days, the patient sudden consciousness deepened again, bilateral pupils dilated, and a computed tomography scan showed increased cerebral hemorrhage and increased ventricular system hemorrhage. Left lateral ventricle puncture drainage and intracranial aneurysm stent-assisted embolization were performed immediately. Digital subtraction angiography revealed successful embolization of the aneurysm (Fig. 3). One month later, CTA revealed good aneurysm embolization (Fig. 4). The patient gradually recovered and was referred to the rehabilitation department for further treatment.

## 3. Discussion and conclusion

Traumatic intracranial aneurysms account for <1% of intracranial aneurysms during peacetime.^[3]^ Traumatic aneurysms near the skull base are more common, especially those originating from the proximal ophthalmic artery.^[4]^ Traumatic anterior cerebral A4 aneurysm is a rare complication of trauma, and its formation may be the result of vascular and cerebral falx trauma.^[5]^ Severe trauma can lead to immediate bleeding and formation of hematoma and pseudoaneurysm.^[6]^ In this case, we report that the patient had no intracranial aneurysm found on CTA within a few hours of admission, and the dissecting aneurysm ruptured 7 days after the trauma. This delayed aneurysm rupture is caused by injury to the intima, internal elastic plate, and middle layer of the blood vessel, while the outer membrane is intact, and the outer membrane forms the aneurysm wall. The rupture time of intracranial aneurysms often varies greatly, mostly 2 to 3 weeks after trauma, and the mortality rate often exceeds 50%.^[7]^ Therefore, timely diagnosis and treatment before aneurysm rupture are very important.^[8]^ CTA or cerebral angiography should be performed immediately after the injury, and CTA or digital subtraction angiography should be reviewed within the next few days. It is safe and feasible to perform stent-assisted embolization after traumatic intracranial aneurysms are discovered.

## Author contributions

**Data curation:** Kai Chen.

**Methodology:** Kai Chen, Wenzhang Luo.

**Resources:** Yu Shi.

**Software:** Tianyu Zhang.

**Writing – original draft:** Yu Shi, Yihang Sui.

**Writing – review & editing:** Changren Huang, Kunyang Bao.
